# Impacts on functional and oncological outcomes of Robotic-assisted Radical Prostatectomy 10 years after the US Preventive Service Taskforce recommendations against PSA screening

**DOI:** 10.1590/S1677-5538.IBJU.2023.0530

**Published:** 2024-03-18

**Authors:** Marcio Covas Moschovas, Abdel Jaber, Shady Saikali, Marco Sandri, Seetharam Bhat, Travis Rogers, Ahmed Gamal, David Loy, Evan Patel, Sumeet Reddy, Maria Chiara Sighinolfi, Bernardo Rocco, Tadzia Harvey, Vincenzo Ficarra, Vipul Patel

**Affiliations:** 1 AdventHealth Global Robotics Institute USA AdventHealth Global Robotics Institute, USA; 2 University of Central Florida USA University of Central Florida (UCF), USA; 3 University of Brescia Data Methods and Statistics Italy Data Methods and Statistics, University of Brescia, Italy; 4 La Statale University ASST Santi Paolo e Carlo Italy ASST Santi Paolo e Carlo - La Statale University, Italy; 5 Università degli Studi di Messina Italy Università degli Studi di Messina, Italy

**Keywords:** Prostatic Neoplasms, Robotic Surgical Procedures, Diagnosis

## Abstract

**Objective:**

In the following years after the United States Preventive Service Task Force (USPSTF) recommendation against prostate cancer screening with PSA in 2012, several authors worldwide described an increase in higher grades and aggressive prostate tumors. In this scenario, we aim to evaluate the potential impacts of USPSTF recommendations on the functional and oncological outcomes in patients undergoing robotic-assisted radical prostatectomy (RARP) in a referral center.

**Material and Methods:**

We included 11396 patients who underwent RARP between 2008 and 2021. Each patient had at least a 12-month follow-up. The cohort was divided into two groups based on an inflection point in the outcomes at the end of 2012 and the beginning of 2013. The inflection point period was detected by Bayesian regression with multiple change points and regression with unknown breakpoints. We reported continuous variables as median and interquartile range (IQR) and categorical variables as absolute and relative percent frequencies.

**Results:**

Group 1 had 4760 patients, and Group 2 had 6636 patients, with a median follow-up of 109 and 38 months, respectively. In the final pathology, Group 2 had 9.5% increase in tumor volume, 24% increase on Gleason ≥ 4+3 (ISUP 3), and 18% increase on ≥ pT3. This translated to a 6% increase in positive surgical margins and 24% reduction in full nerve sparing in response to the worsening pathology. There was a significant decline in post-operative outcomes in Group 2, including a 12-month continence reduction of 9%, reduction in potency by 27%, and reduction of trifecta by 22%.

**Conclusions:**

The increasing number of high-risk patients has led to worse functional and oncologic outcomes. The initial rapid rise in PSM was leveled by the move towards more partial nerve sparing. Among some historical changes in prostate cancer diagnosis and management in the period of our study, the USPSTF recommendation coincided with worse outcomes of prostate cancer treatment in a population who could benefit from PSA screening at the appropriate time.

## INTRODUCTION

In May 2012, the United States Preventive Service Task Force (USPSTF) suggestions against PSA screening drastically reduced the number of patients undergoing PSA test and prostate biopsies. As consequence, several authors have also reported the impacts of these recommendations on prostate cancer treatment (1). Desai and colleagues described a population-based cohort study including 836,282 patients with PCa collected from 2004 to 2018 showing an increase in the incidence of metastatic PCa coinciding temporally with the USPSTF recommendations against PSA screening (2). Similarly, previous studies performed in other centers also detected the same trends highlighting the significant impairment also in the pathology characteristics of treated cancers while comparing outcomes of RARP before and after 2012 (3-5). Obviously, stage migration due to USPSTF suggestions against PSA screening could impact on the characteristics of the treated cancers and could influence treatment related outcomes.

With the evidence described above, considering other historical changes in prostate cancer diagnosis and management through the years, our objective is to analyze the functional and oncologic trends in prostate cancer outcomes ten years after the USPSTF recommendations against PSA screening in patients undergoing robotic-assisted radical prostatectomy (RARP) in a high-volume referral center.

## MATERIALS AND METHODS

**Supplementary Figure 1:**
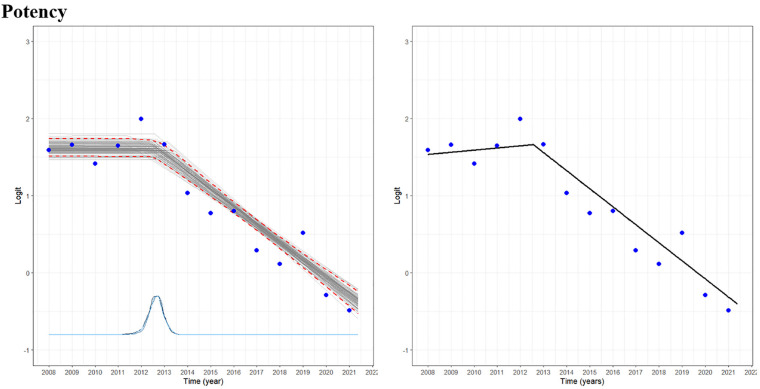
The plots display the results of Bayesian regression with multiple change points (left) and regression with unknown breakpoints (right) for the potency outcome. The left plot illustrates the observed (blue points) and fitted logit of potency rates by years (solid gray lines), along with the 2.5% and 97.5% quantiles (red dashed lines). The distribution displayed at the bottom represents the posterior density of the change point. The right plot shows the fitted broken-line model with the estimated change point.

**Supplementary Figure 2:**
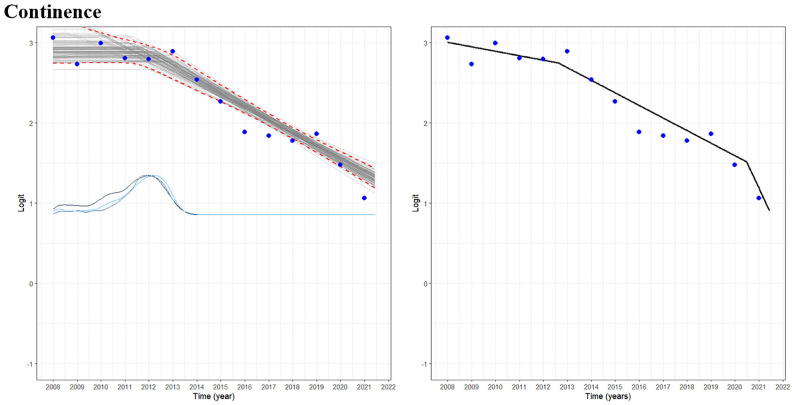
The plots display the results of Bayesian regression with multiple change points (left) and regression with unknown breakpoints (right) for the continence outcome. The left plot illustrates the observed (blue points) and fitted logit of continence rates by years (solid gray lines), along with the 2.5% and 97.5% quantiles (red dashed lines). The distribution displayed at the bottom represents the posterior density of the change point. The right plot shows the fitted broken-line model with the estimated change point.

**Supplementary Figure 3:**
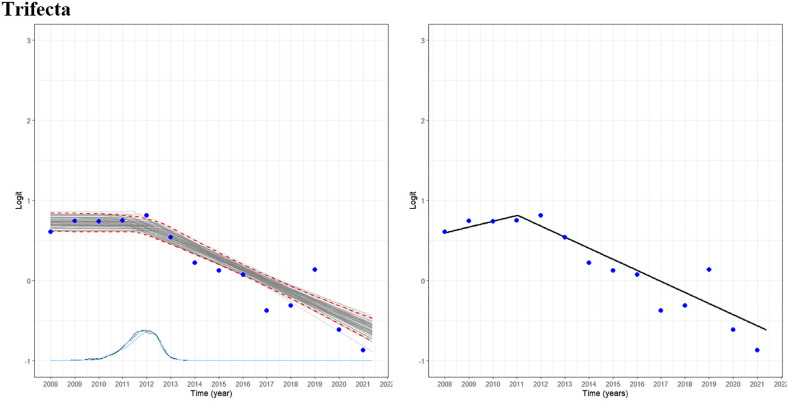
The plots display the results of Bayesian regression with multiple change points (left) and regression with unknown breakpoints (right) for the trifecta outcome. The left plot illustrates the observed (blue points) and fitted logit of trifecta rates by years (solid gray lines), along with the 2.5% and 97.5% quantiles (red dashed lines). The distribution displayed at the bottom represents the posterior density of the change point. The right plot shows the fitted broken-line model with the estimated change point.

**Supplementary Figure 4:**
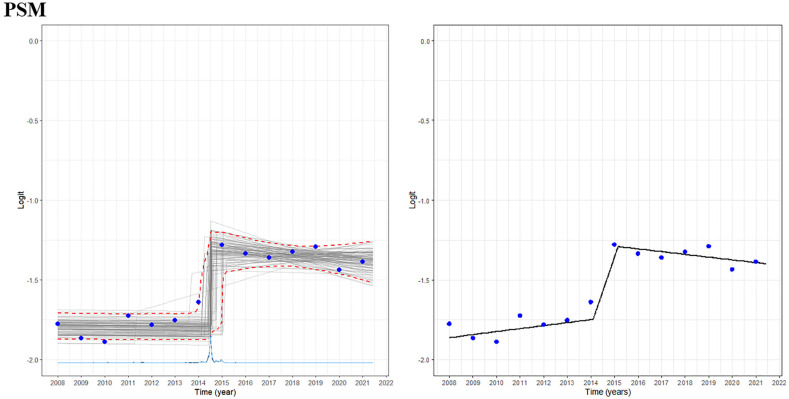
The plots display the results of Bayesian regression with multiple change points (left) and regression with unknown breakpoints (right) for the PSM outcome. The left plot illustrates the observed (blue points) and fitted logit of PSM rates by years (solid gray lines), along with the 2.5% and 97.5% quantiles (red dashed lines). The distribution displayed at the bottom represents the posterior density of the change point. The right plot shows the fitted broken-line model with the estimated change point.

We retrospectively reviewed our prospective IRB-approved (number 237998) prostate cancer registry collecting data of 11,396 consecutive patients who underwent RARP in our center between 2008 and 2021. We selected patients with at least a 12-month follow-up to better evaluate functional and oncological outcomes. Change-point analysis performed using Bayesian regression with multiple change points and regression with unknown breakpoints (6, 7), provided evidence for almost all outcomes of a single inflection point occurring approximately in 2013. For this reason, the cohort was divided into two groups based on this cut-point: before and after the beginning of 2013 (Supplementary Figures 1-4). Therefore, Group 1 included patients from January 2008 to December 2012; and Group 2 included those from January 2013 to December 2021. Group 1 had 4760 patients, and Group 2 had 6636 patients, with a median follow-up of 109 and 38 months, respectively.

We selected the time frame for each group based on the lag time required for the screening recommendations to begin changing clinical practice patterns in our center. Then, we performed a trend analysis for each year by comparing oncological and functional outcomes in the 12 months after following the surgical procedure. Then, we assessed and compared the functional and oncologic outcomes of both groups. Biochemical recurrence (BCR) was described in patients with follow-up ≥ 5 years to avoid describing a curve drop (due to short-term follow-up) and false impressions of BCR reduction after 2017.

### Surgical technique

All patients underwent the same surgical technique with transperitoneal access using four robotic ports and two assistant trocars, according to our previous studies (8-16). The nerve sparing (NS) was divided into grades of preservation (no NS, partial NS, and full NS). Bilateral pelvic lymphadenectomy was performed according to Gleason Score and cancer risk (intermediate and high-risk) (17).

### Step-by step RARP technique:

1. Bladder detachment following anatomical landmarks (umbilical ligaments, pubic bone and deferens);
2. Anterior bladder neck dissection;
3. Posterior bladder neck dissection using the ureters and the longitudinal fibers as landmarks until the Seminal Vesicles (SVs);
4. SVs athermal dissection and control with Hem-o-lok clips;
5. Posterior dissection with athermal technique and nerve-sparing between the Denonvilliers layers;
6. Lateral dissection of the prostate and communication between the lateral and posterior planes;
7. Prostatic arterial pedicles control with Hem-o-lok clips;
8. Apical dissection and DVC control with running suture;
9. Urethra transection and anastomosis with bidirectional running suture;
10. Pelvic lymph node dissection and control with Hem-o-lok clips.

### Postoperative routine and definitions

Five days following surgery, we remove the Foley Catheter and begin rehabilitation for continence and potency. The first PSA is collected six weeks after surgery. In the first year, all patients have routine visits every three to six months, according to the final pathology of each case.

Continence was defined as the capacity of urinary control with no pads used (18). Potency was defined as the capacity of sexual intercourse with or without phosphodiesterase-5 (PDE5) inhibitors (19, 20). BCR was considered when PSA ≥0.2 after RARP. Trifecta was considered when achieving potency, continence, and undetectable PSA levels. The pathology report was described according to the International Society of Urological Pathology (ISUP) and Gleason Scores (21). Tumors classified as ISUP Grade > 3 (> GS 4+3) were defined as aggressive.

### Statistical Analysis

We reported continuous variables as median and interquartile range (IQR) and we compared their distribution between independent groups using the Wilcoxon rank-sum test. We reported categorical variables as absolute and relative percent frequencies using Fisher’s exact test to compare the distribution between groups.

Change point identification in the time trend of cases with the event (potency, continence, BCR, etc.) was performed using two statistical methods: Bayesian regression with multiple change points and regression with unknown breakpoints (6, 7). The two methods are implemented in the mcp and the segmented R packages, respectively (22).

When a change point in the trend was detected, a logistic model with linear segments was estimated; two covariates and their interactions are considered in the model: time as a continuous variable, a binary variable indicating time before/after the change point and the interaction of the two covariates. The two estimated segments of the time trend are then visualized in a plot.

We performed the statistical analysis using STATA 16.1 (StataCorp 2019, College Station, TX, USA), and R 4.1.3 (R Foundation for Statistical Computing, Vienna, Austria). P-values < 0.05 were considered statistically significant.

## RESULTS

Overall, 11,396 patients with a minimum 12-months follow-up after RARP were included in our prospective database.

### Preoperative data

Table-1 compares the preoperative data of two evaluated groups. Notably, patients included in the group 2 resulted significantly younger (p<0.001) and showed a significantly higher median total PSA values in comparison with the group 1 (6 Vs 5 ng/mL). Moreover, after 2012, we observed a 27% reduction in ISUP 1 and a significant increase in ISUP 2, 3, 4, and 5 (p<0.001). Notably, aggressive PCa (ISUP GG >3) were observed in 11% in the group 1 and in 23% in the group 2 (p<0.001).

**Table 1:** Comparison of preoperative demography, nerve sparing degree, and pathological characteristics in the study groups reporting the median value with the interquartile range (IQR) and the number of patients with the percentage. PSA (Prostate Specific Antigen), BMI (Body Mass Index), SHIM (Sexual Health Inventory for Men), AUA (American Urological Association), ISUP (International Society of Urological Pathology).

Parameters		January2008 to December 2012	January 2013 to December 2021	P-value
Total number of patients		4760	6636	< 0.001
Age in years		61	58	< 0.001
(Median, IQR)		(56 - 67)	(64 - 69)	
PSA (ng/mL)		5	6	< 0.001
(Median, IQR)		(3.9 - 6.8)	(4.6 - 8.7)	
BMI (Kg/m^2^)		27.6	27.9	0.001
(Median, IQR)		(25.4 - 30.42)	(25.4- 30.9)	
Preoperative SHIM		21	20	< 0.001
(Median, IQR)		(15 - 25)	(13 - 24)	
Preoperative AUA		7	8	< 0.001
(Median, IQR)		(3 - 12)	(4 -14)	
**Charlson Comorbidity Index n, (%)**				
	0	324 (7)	234 (4)	< 0.001
	1-2	3400 (71)	4049 (61)	
	3-4	995 (21)	2073 (31)	
	≥ 4	41 (1)	280 (4)	
**Biopsy ISUP grade n, (%)**				
	Group 1	2440 (51)	1606 (24)	
	Group 2	1386 (29)	2278 (34)	
	Group 3	447 (9)	1248 (19)	< 0.001
	Group 4	300 (7)	837 (13)	
	Group 5	187 (4)	667 (10)	
**Nerve sparing (NS) degree n, (%)**				
	Bilateral full	2467 (52)	1893 (28)	< 0.001
	Partial	2096 (44)	4739 (71)	
	No nerve sparing	197 (4)	4 (0.1)	
Tumor dimension on pathology report	1.5	1.6	< 0.001
(centimeters)	(1- 2)	(1.2- 2.1)	
Follow-up (months)	109	38	< 0.001
(Median, IQR)	(68- 121)	(24-62)	
**Pathological Grade Group (GrGP), n (%)**	
	GrGP1	1548 (32)	892 (13)	< 0.001
	GrGP2	2091 (44)	2581 (39)	
	GrGP3	726 (15)	1718 (26)	
	GrGP4	124 (3)	234 (4)	
	GrGP5	271 (6)	1211 (18)	
**Pathological Stage, n (%)**				
	pT2	3599 (75)	3784 (57)	< 0.001
	≥ pT3	1161 (25)	2852 (43)	
	Overall PSM, n (%)	681 (14.3)	1345 (20.3)	< 0.001
	PSM on pT2, n (%)	315 (6.6)	379 (5.7)	0.047
	PSM on ≥pT3, n (%)	366 (7.7)	966 (14.6)	< 0.001
Overall Continence achieved, n (%)		4503 (95)	5733 (87)	< 0.001
Overall Potency achieved, n (%)		3170 (67)	2648 (40)	< 0.001
Potency in patients with SHIM ≥ 21, n (%)		2064 (83)	1755 (58)	< 0.001
Potency in patients with SHIM ≥ 21 and full nerve-sparing, n (%)		1333 (88)	898 (76)	< 0.001
Overall Trifecta achieved, n (%)		2527 (54)	2070 (32)	< 0.001

### Final pathology analysis and surgical outcomes

**Figure 1:**
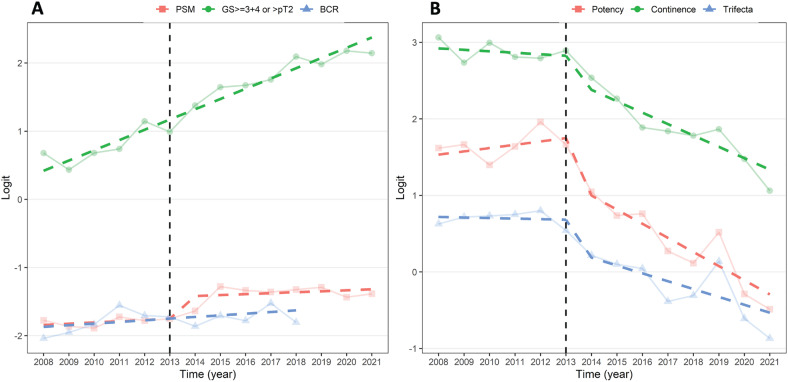
(A) Trend change analysis (logit scale) before and after 2012 showing increasing Gleason ≥7 or ≥ pT3 and increasing positive surgical margins (PSM). Biochemical recurrence (BCR) was considered in patients with follow-up ≥ 5 years to avoid a curve drop and false impression of BCR reduction after 2017. (B) Trend change analysis (logit scale) before and after 2012 illustrating functional outcomes reduction (potency, continence, and trifecta).

Table-1 also illustrates the final pathology and surgical outcomes observed in the two compared groups. Pathological ISUP Grade Groups resulted significantly worse in the group 2 in comparison with group 1 (p<0.001). In details, ISUP GG ≥3 was 48% in group 2 and 24% in group 1. Similarly, pathological non-organ confined tumors (≥pT3) were 43% in the group 2 versus 25% in the group 1 (p<0.001). Interestingly, the overall PSM rate increased from 14.3% reported in group 1 to 20.3% observed in group 2 (p<0.001). Looking at the stratification of PSM rates according to pathologic stage of primary tumor, we observed a statistically and clinically increasing of PSM rate in ≥pT3 tumors (6.7% Vs 13.2% - p<0.001). This translated into a 6% increase in positive surgical margins with an initial rapid increase that was tempered with a surgical adjustment in the amount of nerve-sparing (NS). Indeed, a bilateral full nerve-sparing technique was performed in 52% of RARP until 2012 and only in 28% of procedures performed after 2012 (p<0.001). Figure 1A illustrates a trend change (logit scale) before and after 2012, showing increasing Gleason ≥7 or ≥ pT3 and increasing positive surgical margins (PSM) in different periods.

### Functional outcomes

Comparing groups 1 and 2, there was a significant decline in post-operative outcomes. In details, 12-months urinary continence rate declined significantly from 95% reported in the first group to 87% observed in the group 2 (p<0.001). Similarly, 12-months potency was 67% in the group 1 and only 40% in the group 2 (p<0.001). Therefore, overall Trifecta rate declined from 54% before 2012 to 32% after 2012 (p<0.001).

Figure-1B illustrates a trend change analysis (logit scale) before and after 2012 with evident functional outcomes reduction (potency, continence, and trifecta).

## DISCUSSION

Our study clearly showed that USPSTF recommendations against PSA screening impacted negatively on pathological characteristics of patients who underwent RARP after 2012. As consequence, functional outcomes showed a significant impairment mainly due to the reduction of the nerve-sparing procedures. Notably, we identified a deflection point with changes in the results at the end of 2012 and 2013 illustrated by a trend change analysis (logit scale) coinciding with the USPSTF recommendations against PSA screening in 2012. After this period, we experienced a historical reduction in PSA use on prostate cancer screening by primary doctors and urologists, which was reflected in a significantly increased rate of Gleason 7 or higher, pT3, pT4, and positive surgical margins (PSM). In our experience, we modified our surgical technique to minimize positive surgical margins to address the higher demand for high-risk and invasive tumors while maintaining oncological principles. Consequently, we have seen a reduction in functional outcomes, especially potency recovery, due to a wider dissection needed by these tumors.

When comparing both periods, we detected an 9.5% increase in the median tumor volume reported by the pathology. Therefore, due to the larger tumor burden, we had a significant reduction (24%) in patients undergoing full nerve-sparing with increasing rates of partial nerve-sparing. In this scenario, with more aggressive cancers seen daily in our practice, we described almost 30% reduction in potency outcomes in this group of patients. Similarly, the higher grade and stages at diagnosis were also described in several studies and by Desai and colleagues in a chronological trend analysis after evaluating the Surveillance, Epidemiology, and End Results (SEER) database with more than 830,000 patients (2, 23, 24). Even though we have modified our technique to approach more aggressive tumors, our positive surgical margins (PSM) increased by 6% in patients with pT3 and pT4 stages, while in pT2 continued stable. In this period, the 12-month continence rates were also reduced (by 9%).

Despite the higher demand for aggressive and invasive tumors in our practice, we could maintain our biochemical recurrence percentages constant through the years. We believe that the modifications of our technique to approach these tumors were crucial to maintaining optimal outcomes (12). In our series, we reported BCR in patients with at least five years of follow-up, and curve ends in 2018 to avoid misleading impressions of reduced rates of BCR after this period, which is related to the short-term follow-up and not due to oncological outcomes.

Another factor we believe that may influence the increasing rates of high-grade tumors in the last years is the increasing use of active surveillance (AS) performed in the community with non-standard protocols and follow-up. We recently described our experience comparing patients who underwent RARP at the time of the diagnosis with patients referred to operate in our center after undergoing active surveillance in the community (25). Comparing two groups of 181 patients, matched with a propensity score analysis, we found 16% higher positive surgical margins rates (38% vs. 22%, p=0.001) and a significant increase in biochemical recurrence after surveillance and delayed RARP (HR 4.0; 95%CI 1.4-12; p=0.013). In this study, our main consideration is that we are receiving numerous patients undergoing AS in non-academic centers with non-standard protocols of precise AS indication, follow-up, and treatment plans. Furthermore, we also should consider that in 2013 the group of Johns Hopkins Hospital described a modification in the pathological report and classifications, which consists of five Grade Groups based on the Gleason score (26). In this scenario, the new classification improved the detection rates of clinically significant cancers, and it is challenging to describe the potential impacts of these modifications in the increasing rates of high-risk prostate cancer in our center.

Being a referral center for prostate cancer considered another confounding factor of increasing rates of aggressive tumors during the period of our study should also be considered. With the growing robotic surgery experience and dissemination, most surgeons in the community have been operating low-grade tumors while referring high-risk and challenging cases to these referral centers. In addition, it is important to mention that, for the same reason, there has been a shift through the years in high-risk prostate cancer treatment and management with decreasing rates of radiation therapy and increasing surgical indications by experienced surgeons (27, 28). Radical prostatectomy benefits in high-risk prostate cancer are still debatable, but several retrospective studies have described potential benefits. Recent Randomized Controlled Trials are currently recruiting patients to address these possible questions (29, 30).

The main reason for the USPSTF recommendations against PSA regards the higher rates of overdiagnosis and overtreatment described in some studies, which could potentially benefit patients with low-grade diseases (31, 32). According to their statement (Grade D recommendation), the harms of screening prostate cancer outweigh the benefits. In this scenario, their proposal was quickly incorporated into the urologic clinical practice, and the impacts can be seen in our center and numerous studies performed after that period. The benefits of early prostate cancer detection have been established in the literature for the last 30 years, and PSA screening is a crucial part of this armamentarium. Studies reporting outcomes of prostate cancer screening showed significant reductions in metastasis and mortality before 2012, while studies like ours reported suboptimal oncological outcomes after this period (5, 31-35).

Furthermore, the pillars of these recommendations were the studies performed by Gohagan et al. and Draisma et al., reporting survival rates and possible overtreatment in patients screened for prostate cancer (36-38). However, in a recent reevaluation of these patients, de Vos II and colleagues reported the long-term results (21 years later) of PSA screening showing that after 10 to 12 years, the impacts of these recommendations are evident and patients with 55 to 69 years old from the non-screening group had worse outcomes with higher rates of metastasis and prostate cancer-specific mortality (39).

In the past years, the expansion of focal therapy (FT) also contributed to the increasing rates of high-risk prostate cancer. Some authors described that approximately one-third of these patients undergoing FT would present recurrence and usually have more aggressive tumors (40). However, in our study, we did not include patients undergoing Salvage prostatectomy due to several confounding factors associated with FT, such as type of energy used, FT indication protocol, gland extension (Focal, Hemi, or Whole gland), follow-up routine, and salvage intervention triggers in cases of recurrence (41). Finally, we also should consider the expansion of fusion biopsy as a crucial factor in increasing the detection rates of clinically significant and aggressive cancers. However, this technique has been performed in the last 15 years in a few centers and, despite the improved detection rates, most biopsies in the community are still performed with transrectal ultrasound (TRUS) without fusion (40, 42).

Finally, with the growing body of evidence showing increasing rates of prostate metastasis and aggressive tumors due to lack or reduced applications of prostate cancer screening with PSA (2), we believe that our study is crucial to alert urologists and healthcare community to keep using digital rectal exam (DRE) and PSA as the standard option of prostate cancer screening, especially in countries relying on public health with restricted access to MRI exams and genetic tests.

Despite its strengths, our study is not devoid of limitations, mainly due to the retrospective design and all its inherent risks of bias. We reported a single-center experience with cases done by high-volume surgeons, and despite the comparison group and a trend analysis coinciding with the USPSTF recommendations, surgical outcomes are multifactorial, and it is challenging to establish an exclusive causal factor for these outcomes’ modifications. We also should consider numerous historical changes in prostate cancer diagnosis and management that could influence the increasing rates of aggressive cancers. In addition, the USPSTF reviewed their recommendation and slightly modified it from category D to category C, adding a "sharing decision" in their statement, which also is challenging to measure the impacts on patient care since that year (43). However, to the best of our knowledge, this is one of the largest cohorts reported by a single center comparing outcomes of patients who underwent RARP ten years after the USPSTF recommendations. Therefore, with the data presented in our study and previous articles in the literature, we believe that PSA screening has crucial impacts on functional and oncological results, and urologists and primary care doctors should maintain the screening with PSA and DRE in order to optimize outcomes in patients with prostate cancer.

## CONCLUSION

In the past years, we have witnessed a significant change in the types of patients we treat and the outcomes we are able to deliver. We are seeing younger patients with higher-grade diseases, and the increasing number of high-risk patients has led to worse functional and oncologic outcomes. The initial rapid rise in PSM was leveled by the move towards more partial nerve sparing. Among some historical changes in prostate cancer diagnosis and management in the period of our study, as described in recent populational studies, the USPSTF recommendation coincided with worse outcomes of prostate cancer treatment in a population who could benefit from PSA screening at the appropriate time.
